# Molecular detection of *Toxoplasma gondii* in aborted fetuses of goats in Chattogram, Bangladesh

**DOI:** 10.14202/vetworld.2021.2386-2391

**Published:** 2021-09-14

**Authors:** Tanjila Hasan, Abdul Mannan, Delower Hossain, Azizunnesa Rekha, Md. Monir Hossan, Mohammad Abdul Alim, AHM Musleh Uddin

**Affiliations:** 1 Department of Medicine and Surgery, Faculty of Veterinary Medicine, Chattogram Veterinary and Animal Sciences University, Chattogram 4225, Bangladesh; 2 Teaching and Training Pet Hospital and Research Center, Chattogram Veterinary and Animal Sciences University, Chattogram 4225, Bangladesh; 3 Department of Medicine and Public Health, Faculty of Animal Science and Veterinary Medicine, Sher-e-Bangla Agricultural University, Dhaka 1207, Bangladesh; 4 Upazila Livestock Office, Department of Livestock Services, Farmgate, Dhaka 1215, Bangladesh; 5 Department of Pathology and Parasitology, Faculty of Veterinary Medicine, Chattogram Veterinary and Animal Sciences University, Chattogram 4225, Bangladesh; 6 Department of Surgery and Theriogenology, Faculty of Veterinary, Animal and Biomedical Sciences, Sylhet Agricultural University, Sylhet 3100, Bangladesh.

**Keywords:** aborted fetus, goat, nested polymerase chain reaction, *Toxoplasma gondii*

## Abstract

**Background and Aim:**

*Toxoplasma gondii* is a protozoan parasite that is responsible for the major cause of congenital diseases, abortion, and stillbirth in humans and farm animals. Primary infection in pregnant goats due to *T. gondii* leads to abortion and significant economic losses in the livestock industry. Moreover, very few studies have been performed for the identification of *T. gondii* from aborted fetuses of goats. The study was conducted for the molecular identification of *Toxoplasma gondii* from aborted fetuses of goats in Chattogram, Bangladesh.

**Materials and Methods:**

Twenty aborted fetuses of goats were collected from 52 farms in the study area. A nested polymerase chain reaction (PCR) assay targeting the B1 gene was performed, and a positive sample yield of 197 bp amplified DNA products consistent with *T. gondii*.

**Results:**

The overall prevalence of toxoplasmosis in the aborted fetus of goats was 35%. Heart muscle, liver, brain, and placenta showed positive PCR results. The risk factors related to the does age, presence of cats in farms, and aborted fetus age were found to be statistically significant (p<0.05). Our results showed that *T. gondii* is a major possible causal factor for abortion and reproductive failure in goats. The high prevalence of *T. gondii* infection in aborted fetuses of goats revealed that *T. gondii* could be imperative in causing reproductive failure in goats.

**Conclusion:**

Active or congenital toxoplasmosis was shown by the presence of *T. gondii* in fetal tissues, which is a matter of concern as this parasite has zoonotic significance and causes economic hazards to the livestock industry by causing various reproductive problems. Therefore, proper control measures and strategies are needed to reduce the rate of abortion in goats, ultimately saving the livestock industry.

## Introduction

*Toxoplasma gondii*, an obligate intracellular protozoan parasite with noteworthy zoonotic importance, causes toxoplasmosis in humans and warm-blooded animals [1]. Consumption of raw or uncooked meat and fish containing bradyzoites (water, milk, and vegetables contaminated with oocysts) and transfusion and transplantation of blood and organs, respectively, harboring tachyzoites from patients infected with *T. gondii* are the major sources of *T. gondii* infection in humans [2]. As a final host, cats play an important role in spreading *T. gondii* infection [3]. Therefore, poor hygienic management of farms, climate, presence of cats in farms, consuming raw or uncooked meat and vegetables, and inter-current diseases may act as potential risk factors influencing this disease [4].

Toxoplasmosis is an economically indispensable disease of farm animals, especially goats and sheep [5]. At present, it is considered as a major cause of abortion in goats worldwide [6,7]. The sexual life cycle of *T. gondii* is restricted to cats; however, the asexual cycle depends on humans and other intermediate hosts [8]. Goats are infected by ingesting oocysts excreted through cat feces in the environment and congenital transmission through the placenta. Recently, scientists have reported that tachyzoites might pass over the placenta and infiltrate into the fetus with various consequences, depending on the stage of pregnancy [9,10]. Toxoplasmosis in goats causes early embryonic and fetal death, mummification, abortion, stillbirth, and neonatal death [11]. It is also subsequently associated with calcification and necrosis of the fetal cotyledons and normal intercotyledonary areas [12].

Nowadays, polymerase chain reaction (PCR) amplification of different genes to detect the *T. gondii* from aborted samples is a valuable and reliable technique [6]. A few studies were conducted in Bangladesh on the molecular detection of *T. gondii* in goats and sheep [13]. However, despite the high economic return from goat farming, this enterprise faces several problems on which toxoplasmosis is crucial [14].

Although toxoplasmosis has a huge impact on the health of goats and humans, it was not properly studied in Bangladesh, especially in the Chattogram area. Thus, our study aimed to detect *T. gondii* infection molecularly by nested PCR from aborted goat fetuses in Chattogram, Bangladesh.

## Materials and Methods

### Ethical approval and Informed consent

This study was approved by the department of Medicine and Surgery, Faculty of Veterinary Medicine, Chattogram Veterinary and Animal Sciences University, Chattogram. Moreover, before sample collection, consent from goats’ owner was taken properly.

### Study period and farm selection

A cross-sectional study was conducted from January 2019 to December 2019. Goats of smallholder farms under Chattogram metropolitan area were considered as the reference population. A farm having at least five goats along with the history of abortion was treated as the source population. A total of 52 farms were chosen conveniently representing different surrounding areas of Chattogram metropolitan area. Goats more than 6 months of age beyond selected farms were considered for sample collection. A total of 20 aborted goat fetuses were collected for molecular detection.

### Collection of aborted fetus samples

Eighty seven samples were collected, including heart muscle (20), liver (20), lung (20), brain (20), and placenta (7) from the 20 aborted fetuses of goat. All samples collected were preserved at −20°C until molecular examination.

### Questionnaire survey

A closed-ended questionnaire was used to collect farm demography, farm management, and owners’ information. The questionnaire also included information regarding aborted fetuses and goats, such as age, gestational age, body condition, and presence of cats in and around farm areas. The age of the animal was determined based on dentition and farmers’ information.

### DNA extraction from tissue samples

Genomic DNA extraction from tissue samples (heart, liver, lung, brain, and placenta) was performed using a commercial DNA extraction kit (Favorgen, Favorgen Biotech Corp, Taiwan) following the manufacturer’s protocol. Briefly, 25 mg tissue sample was taken into a sterilized mortar and pestle and ground to make a homogenous mixture using phosphate buffered serum. After centrifugation, cells were lysed and digested with 20 mL proteinase K (10 mg/mL), 4 mL RNase A (100 mg/mL), and 200 mL FATG1 lysis/binding buffer at 70°C for 10 min. Then, 200 mL ethanol (96%~100%) was added, and the mixture was transferred and placed in the FATG Mini column in a 2 mL collection tube and centrifuged at 14,000 rpm for 1 min. Next, the FATG columns were washed twice with washing buffer by centrifugation for 1 min. After, the DNA was eluted from the columns by adding 50 mL elution buffer to the membrane center of the FATG column and stored at −20°C.

### Target genes

The multi-copy B1 gene 197 bp was the PCR target gene evaluated in this study [15]. The B1 gene consists of 35 copies, and it has high sensitivity and specificity among different *T. gondii* strains [15-17]. Primers used for identification of B1 gene: Specific primers were selected according to Wiengcharoen *et al*. [15] for primary and secondary PCR to amplify a portion of the B1 gene and identify the B1 gene (Table-1).

**Table 1:** Selected primer to amplify a portion of B1 gene for the identification of *Toxoplasma gondii*.

Target gene	Primer name	Sequence	Amplification size (bp)	Reference
B1 (primary)	Toxo-F1 Toxo-R1	5´-TCA AGC AGC GTA TTG TCG AG-3´ 5´-CCG CAG CGA CTT CTA TCT CT-3´	-	[15]
B1 (secondary)	Toxo-F2 Toxo-R2	5´-GGA ACT GCA TCCGTT CAT GAG-3´ 5´-TCT TTA AAG CGTTCG TGG TC-3´	197 bp	

### B1 gene amplification by nested PCR and run gel

Nested PCR was performed using a 2720 thermal cycler (Applied Biosystem, USA) in a total reaction volume of 25 mL containing 12.5 mL GoTaq G2 Hot Start Green Master Mix (2×Green GoTaq Reaction Buffer, pH 8.5, 400 mM dATP, 400 mM dGTP, 400 mM dCTP, 400 mM dTTP, and 4 mM MgCl_2_), 1.5 mL of each primer (10 picomoles) derived from B1 gene, 2 mL of the template (sample DNA), and 7.5 mL nuclease free water. For PCR amplification, initial denaturation was performed at 94°C for 30s, followed by 35 cycles of denaturation at 94°C for 15s, annealing at 45°C for 30s, extension at 72°C for 45s, and final extension at 72°C for 10 min. PCR reaction was performed at a temperature similar to the primary PCR for the nested PCR, but the amplification cycles were conducted for 35 cycles. The PCR amplified products were visualized by electrophoresis on agarose gel 1.5% stained with ethidium bromide. Furthermore, the size of DNA fragments was visualized based on a 100 bp DNA ladder under ultraviolet transillumination. The images were captured using a camera.

### Statistical analysis

Data were analyzed using SPSS v.22.0. The normality test of data was performed by the Kolmogorov–Smirnov test [18]. Chi-square test was performed to assess the risk factors [19]. Results were considered significant when p<0.05 [20].

## Results

**Figure-1:**
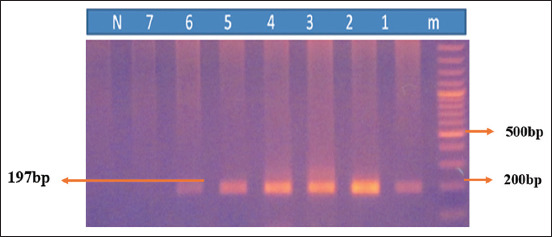
Polymerase chain reaction products amplified using B1 gene. Lane M: 100 bp DNA ladder (Invitrogen), lane N: Negative control, lane 1, 2, 3, 4, 5, and 6: Positive 7 and 8; negative samples.

Twenty aborted fetuses of goats were selected for molecular detection of *T. gondii* by nested PCR. Seven fetuses were positive for the *T. gondii* B1 gene (197 bp) (Figure-1). Our findings revealed that the prevalence of *T. gondii* was 35% in aborted fetuses from goats (Table-2). Here, Figure-1 showed the sizes of the PCR product and distribution of positive cases among different tissue samples from aborted fetuses of goats.

**Table 2:** *Toxoplasma gondii* in aborted fetus of goat based on nested PCR.

Total no. of fetus	*Toxoplasma gondii* nested PCR		Prevalence (%)

	Positive fetus	Negative fetus	
20	7	13	35.00

PCR=Polymerase chain reaction

In this study, 87 tissue samples were tested for *T. gondii* B1 gene detection (Table-3). Seven tissue samples were positive for *T. gondii* (8.1%) (Table-4). Interestingly, heart muscle, liver, brain, and placenta showed positive results in nested PCR. In contrast, lung samples revealed negative results in this study. The positive results were higher in the brain and placenta, followed by the heart muscle. Moreover, the least negative results were found in the lung compared with liver samples.

**Table 3:** *Toxoplasma gondii* in different tissue samples based on PCR assay.

Fetuses	PCR results in different tissue samples					

	Sample no.	Heart muscle	Liver	Lung	Brain	Placenta
Fetus 1	5	+	-	-	-	-
Fetus 2	4	-	-	-	-	NA
Fetus 3	4	-	-	-	-	NA
Fetus 4	4	+	-	-	-	NA
Fetus 5	5	-	-	-	-	-
Fetus 6	4	-	-	-	-	NA
Fetus 7	5	-	-	-	+	-
Fetus 8	4	-	-	-	+	NA
Fetus 9	4	-	-	-	-	NA
Fetus 10	4	-	-	-	-	NA
Fetus 11	5	-	-	-	-	+
Fetus 12	5	-	-	-	-	-
Fetus 13	4	-	-	-	-	NA
Fetus 14	4	-	-	-	-	NA
Fetus 15	4	-	-	-	-	NA
Fetus 16	4	-	-	-	-	NA
Fetus 17	4	-	-	-	-	NA
Fetus 18	5	-	-	-	+	-
Fetus 19	5	-	-	-	-	-
Fetus 20	4	-	+	-	-	NA
Total sample	87	20	20	20	20	7

NA=Tissue not available, PCR=Polymerase chain reaction

**Table 4:** Presence of *Toxoplasma gondii* in different tissue samples from aborted fetus of goats based on nested PCR.

Tissue sample	*Toxoplasma gondii* Nested PCR		Percentage

	Tested sample	Positive sample	
Heart muscle	20	2	10.0
Liver	20	1	5.0
Lung	20	0	0
Brain	20	3	15.0
Placenta	7	1	14.3
Total	87	7	8.1

PCR=Polymerase chain reaction

Age of does, presence of cats in and around farm premises, and age of aborted fetus were found to be statistically significant (p<0.05) variables for the presence of *T. gondii* in aborted fetuses of goats in the Chi-square test (Table-5). The prevalence of *T. gondii* was significantly (p<0.05) higher in does aged 2-3 years compared to >3 years. In addition, the presence of cats in and around farms showed a significantly higher (p<0.05) percentage than the absence of cats. However, aborted fetus age was higher in more than 3.5 months compared to <3.5 months; the difference was statistically significant (p<0.05).

**Table 5:** Distribution of categorical variables among *Toxoplasma gondii* positive aborted fetus of goat.

Variable	Number sampled	Test positive	Prevalence (%)	*df*	χ^2^	p-value
Doe age						
2-3 years	13	5	38.46	1	13.77	p<0.05
>3 years	7	2	28.57			
Presence of cat						
Yes	8	5	62.50	1	8.63	p<0.05
No	12	2	16.67			
Aborted fetus age						
<3.5 months	6	1	16.67	1	7.21	p<0.05
>3.5 months	12	6	50.00		

Here, *df* -Degree of freedom, χ^2^-Chi-square

## Discussion

In our study, the prevalence of *T. gondii* infection in aborted fetuses of goats is 35%. Our findings were similar to that of Unzaga *et al*. [12], who confirmed 36% prevalence in aborted goat fetuses. Similar results were recorded by Pereira-Bueno *et al*. [21], who found 37 out of 106 analyzed fetuses with a prevalence of 34.9%. The prevalence of this study was slightly higher compared with the results of 26.8% from Ghana [22], 27% from Iran [11], and 27.1% from Thailand [23]. Our results are sharply lower than the findings of Chaechi Nosrati *et al*. [10], who showed that the prevalence of *T. gondii* infection was 68.18% based on the molecular nested PCR method [10]. Abu-Dalbouh *et al*. [6] reported that the overall prevalence (%) of *T. gondii* was 41.8% for aborted goats from Jordan. The variation in prevalence is due to variations in health management, feeding and watering systems, nutrition regimens for pregnant animals, biosecurity systems, geographical locations, season, weather, carrier intensification, parasitic genomic diversity, and genetic marker polymorphisms significantly influence the percentage of abortion [24,25].

Several researchers have reported that the brain sample is one of the most susceptible tissues to *T. gondii* infection due to the formation of cysts in the brain [10,21,26,27]. Our results are slightly higher than the findings of Partoandazanpoor *et al*. [11], who detected 8.1% from brain samples. Chaechi Nosrati *et al*. [10] illustrated that *T. gondii* infection was identified in 68.20% of brain tissue samples from aborted ovine fetuses, which is considerably higher than our result. It was observed that fetal brain tissue and placental samples were considered vital tools for diagnosing congenital toxoplasmosis by detecting different *T. gondii* genes by PCR [21].

Risk factors, such as doe age, presence of cats in and around farms, and gestation period influence the prevalence of *T. gondii* in goats [28-30]. There is a significant statistical (p<0.05) difference between prevalence and doe age in this study, and this agrees with the findings of several studies that reported that young animals are more vulnerable to *T. gondii* infections than older animals [31-34]. This may indicate the dominance of maternally acquired antibodies in the early stage of goats, unless re-infected, lessening antibodies as their age rises [29]. Our findings disagreed with that of Saeed *et al*. [30], who reported that older animals are more susceptible than young animals to *T. gondii* infection due to continuous exposure to risk factors for a longer period in older animals.

Our findings revealed a significant relationship between *T. gondii* infections with the presence of cats in farm premises. Recent studies have observed that direct or indirect contact of cats is a prominent risk factor for toxoplasmosis in goats [28,30,35]. Cats are considered a definitive part of the life cycle of *T. gondii* and excrete oocytes of *T. gondii* in their feces, which contaminate water and feed sources and materials. Therefore, there is a high chance of getting infected from contaminated feed and water [30]. Researchers also reported that using surface water sources, including lakes and rivers, as drinking water for animals might be a risk factor for *T. gondii* infections as cats pass out their feces in their (lakes and rivers) contiguity [28].

The frequency of *T. gondii* infection also depends on the age of the aborted fetus. Our findings are similar to those of several studies [5,6,36,37]. The gestation period and immune status of fetuses correlate with the exposure of toxoplasmosis during pregnancy. In early gestational infections, fetal death occurred due to the immature immune system of fetuses [5]. The most common outcome is stillborn during mid-gestational infections, whereas late gestational infections accounted for abortions, stillbirth, or normal offspring [37].

## Conclusion

Our current report on the molecular detection of *T. gondii* infection in goats in Chattogram district, Bangladesh, estimating a higher prevalence. In addition, doe age, presence of cats in farms, and aborted fetus age showed an association with the infection. Our findings will increase the awareness among veterinarians, researchers, and farmers about the epidemiology and distribution of *T. gondii* infection in this region. Further studies are needed to explore more information regarding different genotypes of *T. gondii* and their association with abortion and other reproductive complications in the goat population.

## Authors’ Contribution

TH, AM, and AR: Study design. TH, MMH, and MAA: Performed laboratory experiments. TH, DH, and AHMMU: Drafted the manuscript. AM and MAA: Reviewed and edited the manuscript. All authors read and approved the final manuscript.
